# An Enhanced High-Volume Preparation for Colonoscopy Is Not Better Than a Conventional Low-Volume One in Patients at Risk of Poor Bowel Cleansing: A Randomized Controlled Trial

**DOI:** 10.3389/fmed.2021.654847

**Published:** 2021-03-22

**Authors:** Antonio Z. Gimeno-García, Goretti Hernández, José Luis Baute Dorta, Cristina Reygosa, Raquel de la Barreda, Alberto Hernandez-Bustabad, Carla Amaral, Yaiza Cedrés, Rocío del Castillo, David Nicolás-Pérez, Alejandro Jiménez, Onofre Alarcon-Fernández, Manuel Hernandez-Guerra, Rafael Romero, Inmaculada Alonso, Yanira González, Zaida Adrian, Domingo Hernandez, Laura Ramos, Marta Carrillo, Vanessa Felipe, Anjara Hernández, Consuelo Rodríguez-Jiménez, Enrique Quintero

**Affiliations:** ^1^Servicio de Gastroenterología, Hospital Universitario de Canarias, Instituto Universitario de Tecnologías Biomédicas (ITB), La Laguna, Spain; ^2^Centro de Investigación Biomédica de Canarias (CIBICAN), Departamento de Medicina Interna, Universidad de La Laguna, La Laguna, Spain; ^3^Unidad de Investigación. Hospital Universitario de Canarias, La Laguna, Spain; ^4^UICEC del Complejo Hospitalario Universitario de Canarias, Plataforma SCReN; Servicio de Farmacología Clínica, La Laguna, Spain; ^5^Departamento de Medicina Física y Farmacología, Facultad de Medicina, Universidad de La Laguna (ULL), La Laguna, Spain

**Keywords:** bowel cleansing predictive score, enhanced bowel preparation, hard to prepare patients, high volume bowel preparation, low volume bowel preparation

## Abstract

**Objective:** We tested the hypothesis that an enhanced bowel preparation strategy (EBS) improves colonic cleansing in patients at high risk for inadequate bowel cleansing (HRI).

**Methods:** This prospective randomized clinical trial included consecutive HRI patients referred for outpatient colonoscopy between February and October 2019. HRI was considered if patients scored >1.225 according to a previously validated bowel-cleansing predictive score. HRI patients were randomized (1:1) to a low-volume conventional bowel cleansing strategy (CBS) (1-day low residue diet (LRD) plus 2 L of polyethylene glycol (PEG) plus ascorbic acid) or to an EBS (3-day LRD plus 10 mg oral bisacodyl plus 4 L PEG). The Boston Bowel Preparation Scale (BBPS) was used to assess the quality of cleanliness. Intention-to-treat (ITT) and per protocol (PP) analyses were performed. A sample size of 130 patients per group was estimated to reach a 15% difference in favor of EBP.

**Results:** A total of 253 HRI patients were included (mean age 69.8 ± 9.5 years, 51.8% women). No statistically significant differences were found in the BBPS scale between the two groups in the ITT analysis (CBS 76.8% vs. EBS 79.7%, *P* = 0.58) or PP analysis (CBS 78% vs. EBS 84.3%, *P* = 0.21), risk difference 2.9% (95% CI−7.26 to 39.16) in the ITT analysis, or risk difference 6.3% (95% CI−3.48 to 16.08) in PP analysis. No differences in preparation tolerance, compliance, adverse effects, or colonoscopy findings were found.

**Conclusion:** EBS is not superior to CBS in hard-to-prepare patients. (EUDRACT: 2017-000787-15, NCT03830489).

**Clinical Trial Registration:** www.ClinicalTrials.gov, identifier NCT03830489.

## Introduction

Colonoscopy is the gold standard for the diagnosis of colorectal neoplasia and is currently the technique of choice for both the diagnosis and screening of colorectal cancer, reducing its incidence and mortality (1). Quality in colonoscopy is critical to improve its effectiveness (2, 3). The cecal intubation rate and adenoma detection rate are the main quality factors and are directly linked to cleansing quality. Poor bowel preparation not only has a negative effect on these indicators but is also associated with technical difficulties, risk of complications, increased examination times, and the need for subsequent colonoscopies and ultimately raises costs. Multiple risk factors for poor colon cleansing have been described (4–6). A recent study carried out in a large cohort of consecutive patients scheduled for outpatient colonoscopy who received different split-dose bowel preparations (either low-volume or high-volume preparations) found that a bowel-cleansing predictive score (BCPS) that included comorbidities (mainly diabetes mellitus), antidepressant intake, chronic constipation and pelvic or abdominal surgery were predictive factors for poor bowel cleansing. This predictive model showed an acceptable discrimination between adequate and poor bowel preparation (area under the curve, AUC = 0.70-0.72) (5). Although it has not been demonstrated in clinical practice, this type of model might help to tailor the proper bowel cleansing protocol for each patient.

There is large evidence that low-volume bowel preparation regimens are as high-volume ones in non-selected population (7). However, the current evidence in hard to prepare patients is scarce. Although, one randomized controlled trial carried out in patients with a high risk of poor bowel cleansing (8) (specifically with past history of poor bowel preparation) showed that a high-volume enhanced protocol based on 4 L polyethylene glycol (PEG), bisacodyl and 3 days of a low residue diet (LRD) was better than a low-volume-based regimen (2 L PEG plus ascorbic acid and bisacodyl and 3 days of LRD), the same results would not necessarily be expected for other groups of patients with high risk factors (HRI) for poor bowel preparation.

The hypothesis of this study was that in HRI patients determined by the BCPS (score >1.225), an enhanced cleansing protocol is better than a conventional low-volume-based regimen, as it works in patients with a past history of poor bowel preparation.

## Materials and Methods

### Design and Setting

This prospective randomized trial was conducted at the Open Access Endoscopy Unit of the Hospital Universitario de Canarias between February 2019 and October 2019. This hospital is a tertiary referral hospital that provides health care to ~400,000 inhabitants of the northern part of Tenerife Island. The endoscopy unit has an annual output of ~6,000 outpatient colonoscopies, 3,000 of which are performed during morning sessions.

The Ethics Committee approved the study protocol in July 2017. The trial was registered in the Agencia Española del Medicamento (August 2, 2017), European Union Clinical Trial Register (EUDRACT 2017-000787-15) in February 2017 and ClinicalTrials.gov (NCT03830489) in February 2019. The first patient was included in February 2019, and the last patient was included in October 2019. All authors had access to the study data and reviewed and approved the final manuscript.

The study has been reported in accordance with the Consolidated Standards of Reporting Trials (see the CONSORT checklist in online-only Supplementary Material).

### Patients

Patients older than 18 years undergoing outpatient colonoscopy in the morning were considered for inclusion. The BCPS was calculated for every outpatient scheduled for a colonoscopy during the inclusion period. The BCPS is composed of 4 criteria (Table 1). Details of the design and validation of this score have been previously reported (5). For the purpose of the study, only patients with a BCPS score >1.225 were included.

**Table 1:** Validated bowel cleansing predictive score.

**Predictor factor of inadequate bowel preparation**	**Score**
Comorbidity^a^	4
Tricyclic antidepressants	1.705
Chronic constipation^b^	1.225
Abdominal or pelvic surgery	0.606

a *Diabetes mellitus, stroke, liver cirrhosis, chronic kidney disease (glomerular filtration rate < 60 mL/min)*.

b *<3 bowel movements/week and at least one of the following: straining, hard stools defined as Bristol scale 1 or 2 and incomplete evacuation*.

The exclusion criteria were as follows: past history of poor bowel cleansing because, although it is a well-known predictor of poor bowel preparation, these patients may benefit from enhanced bowel preparation (8), bowel obstruction, megacolon, intestinal perforation, poorly controlled arterial hypertension (arterial systolic blood pressure >180 mmHg and/or arterial diastolic blood pressure >100 mmHg), congestive heart failure, NYHA III-IV acute liver failure, end-stage renal failure (dialysis or predialysis), pregnancy, lactation, dementia with difficulties following the instructions, known hypersensitivity reaction to the components of the drug, diagnosis of phenylketonuria, diagnosis of glucose-6-phosphate dehydrogenase deficiency, colectomy of more than one segment, and refusal to participate.

### Procedures Before Colonoscopy

Four researchers not involved in the colonoscopy procedures explained the purpose of the study, verified the inclusion and exclusion criteria, obtained informed consent and completed a data collection sheet. Oral and written instructions about the bowel cleansing preparation were also given according to the allocation group. The patients were advised to complete a diet register for 1 or 3 days before the colonoscopy appointment, depending on the allocation group.

### Randomization and Group Descriptions

The randomization sequence was computer generated in a 1:1 sequence by a statistician of the Research Unit of our hospital. Sealed randomization envelopes were used. Patients with a BCPS score >1.225 were randomized to one of the following two groups:

1. Enhanced bowel preparation strategy (EBS): patients assigned to this group received a LRD 3 days before the colonoscopy. They also took 2 tablets of bisacodyl (10 mg) at 19:00 and 2 L of PEG (8 sachets) 12 h before the appointment and another 2 L of PEG (8 sachets) 5 h before the appointment for the colonoscopy.
2. Conventional bowel preparation strategy (CBS): patients assigned to this group were prepared the day before the examination with a LRD and 1 L of PEG with ascorbic acid (PEG+Asc) (one envelope A and one envelope B) 12 h before the colonoscopy appointment and 1 L of PEG with ascorbic acid (one envelope A and one envelope B) 4 h before the colonoscopy appointment. Patients were recommended to drink 500 ml more water after ingesting the bowel solution.

The LRD recommended to both groups was specifically designed by an endocrinologist specialized in nutrition.

### Colonoscopy Procedures

Colonoscopies were scheduled in the morning session. Three nurses involved in the study who were blinded to the allocation group collected information regarding tolerance, satisfaction, difficulties drinking the bowel solution, willingness to follow the same bowel preparation in the future, incidents and side effects. Patients returned the food record sheet on the day of the colonoscopy.

Colonoscopies were performed by five experienced endoscopists. The whole endoscopy team was blinded to the patient allocation group. The Boston Bowel Preparation Scale (BBPS) (9) was registered in the colonoscopy report together with the colonoscopy findings (number, size, and morphological characteristics of any polyp). The endoscopists passed the BBPS Educational Program by obtaining a score ≥ 3 (5).

### Variables Collected

#### Patient Variables

Variables collected included demographic details; indication for colonoscopy; educational level (higher or lower than high school); personal history of colonic polyps or colorectal cancer; comorbidities (diabetic patients under pharmacological treatment; cirrhosis diagnosed by clinical, imaging or analytical criteria; stroke; or chronic kidney disease defined as a renal glomerular filtration rate <60 mL/min); history of abdominal or pelvic surgery; constipation (<3 bowel movements/week and at least one of the following: straining, hard stools defined as Bristol scale 1 or 2, and incomplete evacuation) (10); and medication (treatment with tricyclic antidepressants, opioids or calcium antagonists).

#### Variables Collected on the Day of Colonoscopy

The following variables were collected: the elapsed time between the last intake of solution and the beginning of the colonoscopy; willingness to follow the same preparation protocol in the future (11); any difficulty in following the bowel preparation instructions; level of satisfaction (12); volume intake categorized as ≥75% or <75% of the bowel preparation; adverse effects and incidents of the preparation protocol according to the American Society of Gastrointestinal Endoscopy lexicon (13); BBPS score (global and by colonic segment); cecal intubation rate; and complications related to the colonoscopy (perforation or postpolypectomy bleeding requiring hospitalization). The withdrawal time from the cecum was recorded using a stopwatch; the watch was stopped when any biopsy or therapeutic technique was required and then resumed after the completion of these procedures. The amount of water used for lavage during each examination was also quantified by counting the number of 50-mL water syringes used. The number of polyps and their sizes and locations were also recorded.

### Outcomes

#### Primary Outcome

The primary outcome was the rate of adequate bowel cleansing assessed by the BBPS (9). This validated scale ranges from 0 to 3 points per segment (proximal, transverse and distal colon). For the intention-to-treat (ITT) analysis, in complete colonoscopies, bowel cleansing was adequate when each of the colon segments were assessed and scored ≥2 points. Bowel cleansing was considered inadequate when the score in at least one of the segments was <2 points. In incomplete colonoscopies, bowel cleansing was considered inadequate when a segment was not assessed.

#### Secondary Outcomes

Adherence to the bowel cleansing instructions was tested by a personal food record (14) when the volume of solution ingested was ≥75%.

The level of satisfaction and difficulties following bowel preparation were assessed using a 5-point subjective scale (12). Willingness to repeat the same bowel cleansing protocol in the future was assessed as a dichotomous variable (yes/no) (11).

Adverse effects and incidents were assessed by asking the patients about events potentially related to bowel preparation, such as nausea, vomiting, bloating, and abdominal pain.

### Statistical Analysis and Sample Size

In a previous study conducted in our unit, 25% of patients who attended a colonoscopy had a score >1.225 on the BCPS (5). For the present study, we estimated a clinically relevant difference in the proportions of the rate of adequate bowel preparation between EBS and CBS of at least 15% in favor of EBS. Assuming a type I error of 5%, a power of 80%, and considering a dropout rate of 15%, 130 participants were needed to be included per group. Sample size was calculated with GRANMO v. 7.12 (IMIM, Barcelona, Spain. https://www.imim.cat/ofertadeserveis/software-public/granmo/).

The two groups were compared using the chi-square statistic for categorical variables and Student's *t*-test for continuous variables. Intention-to-treat (ITT) and per-protocol (PP) analyses were conducted.

All available variables likely associated with the outcome were analyzed using univariate logistic regression. Variables that achieved at least *P* < 0.05 were entered into the multivariate logistic regression. The results are expressed as odds ratios (ORs) with 95% confidence intervals (CIs). *P-*values of <0.05 were considered to be statistically significant. The Statistical Package for the Social Sciences v. 20.0 (SPSS, Inc., Chicago, Illinois, USA) was used for all statistical analyses.

## Results

**Figure 1:**
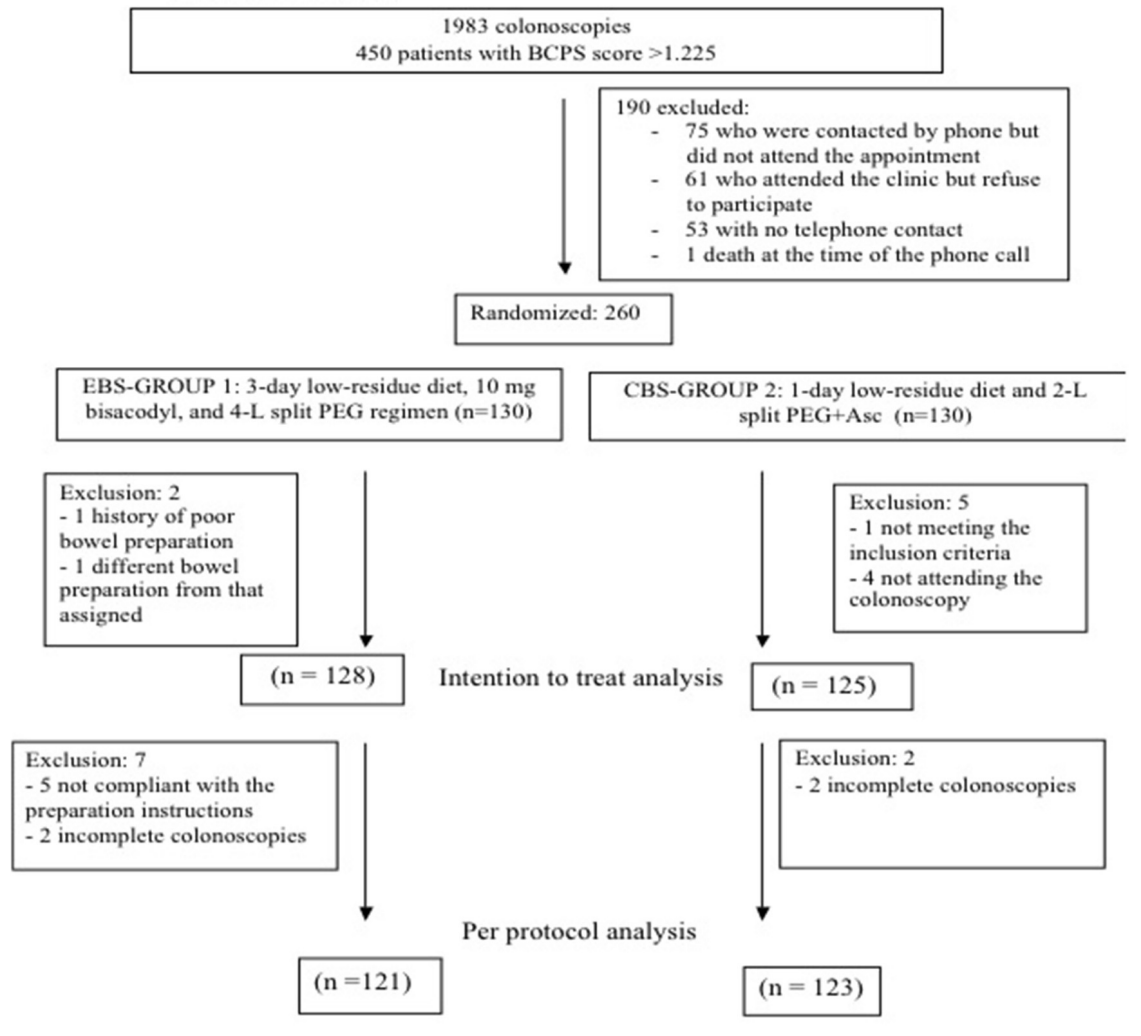
Flow chart.

During the study period, a total of 1,983 patients were scheduled for a colonoscopy in the morning shift. Overall, 450 (22,6%) patients had a BCPS score >1.225 and were eligible for the study. An appointment was scheduled for 396 patients, of whom 75 did not attend the appointment and 61 refused to participate. Finally, 260 patients were randomized, and 130 patients were assigned to each group. Two patients were excluded after inclusion in the EBS group, and 5 were excluded in the CBS group. Finally, 128 patients and 125 patients were included in the EBS and CBS groups, respectively (Figure 1). There were no statistically significant differences regarding baseline characteristics between groups (Table 2).

**Table 2:** Basal characteristics of patients.

**Demographic and clinical variables**	**EBS^a^** **(*n* = 128)**	**CBS^b^** **(*n* = 125)**	***P***
Age (mean ± SD)	70.3 ± 9.63	69.3 ± 9.52	0.41
Sex (male), *n* (%)	61 (47.7)	62 (49.6)	0.76
BMI^c^ (mean ± SD)	29.33 ± 4.82	28.98 ± 5.43	0.60
Education,^d^ *n* (%)	23 (18.0)	31 (24.8)	0.19
FDRs,^e^ *n* (%)	20 (15.6)	23 (18.4)	0.56
Personal history of adenoma, *n* (%)	40 (31.3)	40 (32.0)	0.90
**Comorbidity**, ***n*** **(%)**			
Diabetes	99 (77.3)	101 (80.8)	0.50
Stroke	10 (7.8)	8 (6.4)	0.66
Cirrhosis	4 (3.1)	1 (0.8)	0.37
Chronic renal failure	16 (12.6)	17 (13.6)	0.81
Hypertension	54 (42.2)	47 (37.6)	0.17
Constipation	23 (18.0)	24 (19.2)	0.80
Abdominal/pelvic surgery	62 (48.2)	56 (44.8)	0.56
**Medical treatment**, ***n*** **(%)**			
Opioids	7 (5.5)	11 (8.8)	0.30
Calcium antagonists	21 (16.4)	23 (18.4)	0.68
Antidepressants	7 (5.5)	5 (4.0)	0.58
**Indications**, ***n*** **(%)**			
Anemia	24 (18.8)	24 (19.2)	0.93
Rectal bleeding	9 (7.0)	16 (12.8)	0.12
Postpolypectomy surveillance	35 (27.3)	33 (26.4)	0.87
Average-risk population screening	34 (26.6)	27 (21.6)	0.36
Familial colorectal cancer screening	5 (3.9)	8 (6.4)	0.37
Change in bowel habits	15 (11.7)	9 (7.2)	0.22
Other	6 (4.7)	8 (6.4)	0.55
BBPS^f^ at index colonoscopy	1.34 ± 1.411	1.57 ± 1.536	0.22

a *EBS, enhanced bowel preparation strategy: 4-L split-dose polyethylene glycol (PEG) regimen*.

b *CBS, conventional bowel preparation strategy: 2-L split-dose PEG plus ascorbic acid (PEG + Asc) regimen*.

c *Body mass index*.

d *Education higher than high school*.

e *First-degree relatives with colorectal cancer*.

f *Boston Bowel Preparation Scale*.

### Quality of Bowel Cleansing

There was an inverse correlation between the BCPS and bowel cleansing assessed by the quantitative BBPS (Spearman's rank correlation coefficient = 0.218, *P* < 0.001). In the ITT analysis, globally adequate bowel preparation was achieved in 79.7% of patients assigned to EBS (95% CI [70.1–86.2]) and in 76.8% of those receiving CBS (95% CI [72.0–88.0]) (OR 1.2, 95% CI [0.65–2.16], *P* = 0.58). The data for the PP analysis were 84.3%, 95% CI [75.3–90.0] for the EBS and 78%, 95% CI [73.4–89.1] for the CBS (OR 1.5, 95% CI [0.79–2.89], *P* = 0.21). There were no statistically significant differences in bowel quality *per* segment (Table 3).

**Table 3:** Comparison of adequate bowel cleansing between study groups.

**Global and per-segment adequate cleansing**	**EBS^a^**	**CBS^b^**	***P***
**Intention-to-treat analysis**	**(*n* = 128)**	**(*n* = 125)**	
Global BBPS^c^ score ≥ 2 per segment—no. (%)	102 (79.7)	96 (76.8)	0.58
Left BBPS score ≥ 2—no. (%)	113 (88.3)	108 (86.4)	0.65
Transverse BBPS score ≥ 2—no. (%)	104 (82.5)	93 (79.5)	0.54
Right BBPS score ≥ 2—no. (%)	110 (85.9)	108 (87.1)	0.79
Mean BBPS in the whole colon (mean, SD)^d^	6.05 (2.118)	5.66 (2.314)	0.17
Mean BBPS in the left colon (mean, SD)	2.1 (0.719)	2.02 (0.788)	0.41
Mean BBPS in the transverse colon (mean, SD)	2.05 (0.782)	1.98 (0.801)	0.48
Mean BBPS in the right colon (mean, SD)	1.93 (0.771)	1.82 (0.784)	0.28
**Per-protocol analysis**	**(*n* = 121)**	**(*n* = 123)**	
Global BBPS score ≥ 2 per segment—no. (%)	102 (84.3)	96 (78.0)	0.21
Left BBPS score ≥ 2—no. (%)	110 (90.9)	106 (86.2)	0.25
Transverse BBPS score ≥ 2—no. (%)	108 (89.3)	107 (87.0)	0.59
Right BBPS score ≥ 2—no. (%)	104 (86.7)	93 (79.5)	0.14
Mean BBPS in the whole colon (mean, SD)	6.29 (1.823)	5.70 (2.315)	0.028
Mean BBPS in the left colon (mean, SD)	2.17 (0.624)	2.02 (0.794)	0.125
Mean BBPS in the transverse colon (mean SD)	2.12 (0.678)	1.98 (0.804)	0.121
Mean BBPS in the right colon (mean SD)	2.02 (0.673)	1.82 (0.784)	0.04

a *EBS, enhanced bowel preparation strategy: 4-L split-dose polyethylene glycol (PEG) regimen*.

b *CBS, conventional bowel preparation strategy: 2-L split-dose PEG plus ascorbic acid (PEG+Asc) regimen*.

c *BBPS: Boston Bowel Predictive Scale*.

d *Mean, SD: mean ± standard deviation*.

In the ITT analysis, the 95% CI of the difference in proportions for the rate of adequate bowel preparation was −7.26 to 39.16%, whereas in the PP analysis it was −3.48 to 16.08%, confirming that the enhanced 4 L PEG preparation was not superior to the conventional bowel preparation. In addition, the mean total or per-segment BBPS scores were not significantly different between the groups in the ITT analysis (mean total BBPS score, *P* = 0.17; mean BBPS score in the left colon, *P* = 0.41, mean BBPS score in the transverse colon, *P* = 0.48; mean BBPS score in the right colon, *P* = 0.28). The whole and proximal colon quality quantitative scores in the PP analysis were better in patients assigned to the EBS group than in those assigned to the CBS group (*P* = 0.028 and *P* = 0.04, respectively) (Table 3).

### Bowel Preparation and Colonoscopy Findings

Cecal intubation was achieved in 94.2 and 90.2% of patients assigned to the EBS and CBS groups, respectively (Table 4). There were no statistically significant differences between groups regarding the number of 50 mL syringes used for lavage or the withdrawal time. Regarding neoplastic findings, the colorectal cancer detection rate, polyp detection rate, adenoma detection rate, diminutive polyp detection rate, diminutive adenoma detection rate and the number of polyps or adenomas per patient were comparable between groups. No serious adverse effects were derived from colonoscopy procedures.

**Table 4:** Colonoscopy findings.

**Colonoscopy findings**	**EBS^a^** **(*n* = 121)**	**CBS^b^** **(*n* = 123)**	***P***
Cecal intubation rate—no. (%)	114 (94.2)	111 (90.2)	0.25
Lavage, mL—mean (SD)	131.8 (110.13)	114.7 (129.02)	0.41
Withdrawal time, min—mean (SD)	9.6 (2.91)	10.0 (3.54)	0.39
Colorectal cancer detection rate—no. (%)	6 (5)	3 (2.4)	0.3
Polyp detection rate—no. (%)	56 (46.3)	53 (43.1)	0.62
Adenoma detection rate—no. (%)	52 (43)	48 (39)	0.43
Diminutive polyp detection rate—no. (%)	44 (36.4)	42 (34.1)	0.52
Diminutive adenoma detection rate—no. (%)	41 (339)	39 (31.7)	0.68
Number of polyps per patient—mean (SD)^c^	1.30 (2.47)	1.31(2.15)	0.97
Number of adenomas per patient—mean (SD)	1.04 (1.97)	1.07(1.75)	0.92
Adverse effects—no. (%)	0	0	–

a *EBS, enhanced bowel preparation strategy:4-L split-dose polyethylene glycol (PEG) regimen*.

b *CBS, conventional bowel preparation strategy: 2-L split-dose PEG plus ascorbic acid (PEG + Asc) regimen*.

c *Mean (SD): mean ± standard deviation*.

### Tolerance, Acceptance and Willingness to Receive the Same Bowel Preparation in the Future

Overall, 116 (97.5%) and 113 (99.1%) patients were compliant with the diet recommendations in the EBS and CBS, respectively. Regarding bowel preparation adherence, only 2 patients in the EBS took <75% of the bowel preparation.

Although no adverse effects were reported, incidents occurred in 21.9% and 18.4% (*P* = 0.49) of patients in the EBS and CBS, respectively (Table 5). Nausea was the most frequent incident and was reported by 13% of the patients in both groups.

**Table 5:** Tolerance, acceptance, and willingness to repeat the same bowel preparation.

	**EBS^a^** **(*n* = 128)**	**CBS^b^** **(*n* = 125)**	**OR (95% CI)**	***P***
**Patient-reported outcomes**, ***n*** **(%)**				
Incidents	28 (21.9)	23 (18.4)	0.8 0.8 (0.44 to 1.49)	0.49
Good or excellent satisfaction	115 (89.8)	109 (87.2)	0.6 0.6 (0.34 to 1.18)	0.38
Few or no difficulties	126 (98.4)	123 (98.4)	1.0 1.0 (0.14 to 7.39)	1.00
Willingness to repeat	116 (91.3)	110 (88.0)	0.8 0.8 (0.28 to 2.09)	0.38

a *EBS, enhanced bowel preparation strategy: 4-L split-dose polyethylene glycol (PEG) regimen*.

b *CBS, conventional bowel preparation strategy: 2-L split-dose PEG plus ascorbic acid*.

*LRD, low-residue diet; OR, odds ratio; CI, confidence interval*.

In general, the satisfaction level was high for both bowel preparations; most patients had few difficulties taking the assigned solution, and most of them were willing to repeat the same preparation in the future (Table 5).

### Variables Associated With Poor Bowel Preparation

Univariate and multivariate analyses were carried out to assess variables associated with poor bowel cleansing. The patients included in the ITT analysis were entered into both analyses. Supplementary Table 1 shows the univariate analysis. Only difficulties following bowel preparation (OR 12.06, 95% CI [1.15–126.30]) and suffering a stroke (OR 3.22, 95% CI [1.17–8.85]) were independently associated with poor bowel cleansing (Supplementary Table 2).

## Discussion

The optimal bowel preparation in hard-to-prepare patients is currently unknown, and recommendations from scientific societies have low evidence-based support (15, 16).

In this randomized controlled trial, we showed that an enhanced bowel preparation based on a 3-day LRD, 10 mg of bisacodyl and a 4 L PEG solution was not more effective than a conventional low-volume bowel preparation based on a 1-day LRD and 2 L PEG+Asc. This result was unexpected to us because we designed a superiority analysis in favor of the large volume-based preparation. In a recent randomized controlled trial carried out by our group (8) in patients with a high risk of poor bowel preparation (specifically, a past history of poor bowel preparation) the same intensive large bowel preparation had a higher efficacy than a low-volume preparation protocol (adequate preparation: 81.1% vs. 67.4%, difference in proportions: 13.7%, 95% CI 3.13%−24.27%). Although the results of both studies might seem contradictory, a past history of poor bowel preparation has been stated to be one of the most powerful predictors of inadequate bowel preparation in a future colonoscopy and can be considered objective proof of difficulty obtaining adequate bowel cleansing (17, 18). Conversely, other risk factors for poor bowel preparation, such as those that make up the BCPS, may not be a guarantee for difficulty in achieving adequate bowel cleansing. Although, some of these factors have been widely recognized as predictors of bowel cleansing failure, most patients who meet these criteria would currently have adequate bowel cleansing (5) and it may explain the different results obtained in both studies. In an observational study carried out in 1,073 outpatients, antidepressant use, comorbidities, past history of abdominal or pelvic surgery, and chronic constipation were independent predictors of inadequate bowel preparation and were used to develop and validate the predictive model used in the present study (5). The area under the curve (AUC) of the BCPS in the development cohort and the validation cohort in this study was 0.72 (95% CI, 0.69–0.75) and 0.70 (95% CI, 0.65–0.74), respectively.

To our knowledge, this is the first randomized controlled trial to assess the effect of enhanced bowel preparation in patients with a high risk of poor bowel cleansing following a predictive model score.

Many randomized studies have compared high-volume bowel preparations with low-volume bowel preparations in hard-to-prepare patients (19–21). These studies were carried out in specific populations, such as patients with spinal cord injury (19), patients with chronic constipation (21) and patients with a colectomy (20). Two of them compared a large bowel 4 L PEG preparation with a low volume 2 L PEG preparation with bisacodyl (20, 21), and one study compared 4 L PEG with sodium phosphate (19). In these studies, there were no significant differences in cleansing efficacy between the two regimens. However, none of them actually used enhanced cleansing protocols such as the one used in the present study (high-volume preparation plus adjuvant plus 3-day LRD) but only employed conventional high-volume preparations.

A novelty of the present study was the first ever use of a predictive model tested in a population other than the one used to design the model. Three predictive models for assessing poor bowel cleansing have been developed so far (4–6), two of which were validated in the same study (4, 5). These models have several flaws such as the lack of internal or external validation, the fact that some patients were prepared the day before the examination, the inclusion of non-compliant patients, the use of a non-validated bowel cleansing scale during the colonoscopy. preparation protocols differed between the centers, and the inclusion of patients with a past history of poor bowel preparation Unlike, the two other predictive models the predictive score used in the present study overcame most of these limitations since the patients took at least part of the preparation on the same day of the examination, we used a validated bowel cleansing scale, and we excluded those patients with a past history of poor bowel preparation (5).

Finally, we are aware that our study has some limitations. First, this was a single-center study, and our results should be replicated by other groups and in future multicenter prospective studies. Second, the inclusion criterion of a BCPS score >1.225 was made based on an uncontrolled observational study. However, the variables included in the BCPS are widely recognized as predictive factors for poor bowel cleansing. Third, since before the examination, the patients included in the study attended a consultation with a physician who explained the purpose of the study and bowel preparation, we believe that our bowel quality results may have been influenced by this educational intervention. However, both study arms received the same intervention.

Finally, although this study suggests that administering a greater volume does not result in better bowel cleansing, the results may not be generalized to the rest of the low-volume agents other than PEG+Asc. It is also unclear if adding more volume of bowel solution (i.e., 6 L of PEG) or increasing the low-residue diet days should have an additional benefit. Rescue strategies such as the administration of additional solution just before the examination based on the effluent description by the patients could be an alternative to reduce the percentage of re-scheduled colonoscopies for poor bowel cleansing. In conclusion, this study demonstrated that the EBS based on a 3-day LRD, 10 mg oral bisacodyl and 4L PEG is not better than a conventional protocol with a 1-day LRD and 2 L PEG+Asc in patients with risk factors for poor bowel preparation, excluding those with a previous suboptimal bowel preparation. Further studies are warranted to test other enhancing cleansing protocols in this subgroup of patients.

## Data Availability Statement

The raw data supporting the conclusions of this article will be made available by the authors, without undue reservation.

## Ethics Statement

The studies involving human participants were reviewed and approved by Comité de Ética e Investigación Clínica. Hospital Universitario de Canarias. The patients/participants provided their written informed consent to participate in this study.

## Author Contributions

AG-G and EQ: final approval. AG-G, EQ, and AH-B: drafting the manuscript. AJ, OA-F, AG-G, MH-G, and CR: analysis and/or interpreting data. GH, DN-P, VF, MC, RR, YG, ZA, IA, LR, DH, JB, AH, RB, CA, YC, and RC: collecting data. AG-G, EQ, and CR: planning the study and/or conducting the study. All authors have contributed substantially to this study: design, data collection, analysis of data, and preparation of the manuscript.

## Conflict of Interest

The authors declare that the research was conducted in the absence of any commercial or financial relationships that could be construed as a potential conflict of interest.

## Abbreviations

EBS: enhanced bowel preparation strategy
HRI: high risk of inadequate bowel cleansing
CBS: conventional bowel cleansing strategy
LRD: low residue diet
ITT: intention-to-treat
PP: per protocol
PEG: polyethylene glycol
BCPS: bowel-cleansing predictive score
OR: odds ratios
CI: confidence Interval
Asc: ascorbic acid.
