# Supplementary material for: An Enhanced High-Volume Preparation for Colonoscopy Is Not Better Than a Conventional Low-Volume One in Patients at Risk of Poor Bowel Cleansing: A Randomized Controlled Trial
Source: Front Med (Lausanne). 2021 Mar 22;8:654847. doi: 10.3389/fmed.2021.654847 (PMC8019748; doi:10.3389/fmed.2021.654847)
Supplement: Supplementary file 2 [file Table_1.doc]

Supplementary table 1. Univariate analysis. Variables associated with poor bowel cleansing.

|  | **BBPS<2^a^**  **(n=55)** | **BBPS≥2^b^**  **(n=198)** | **OR (95% CI) P** |
| --- | --- | --- | --- |
| Age, (mean, SD) | 72.1(8.7) | 69.1(9.6) | 1.04(1.001-1.07) 0.04 |
| Sex (male), n (%) [95% C] | 28 (50.9) | 95 (48.0) | 1.4(0.75-2.49) 0.7 |
| BMI^c^, (mean, SD) | 29.1 (6.5) | 29.2 (4.7) | 1.0(0.94-1.06) 0.97 |
| Education^d^, n (%) | 7 (12.7) | 47 (23.7) | 0.5(0.19-1.10) 0.08 |
| FDRs^e^, n (%) | 9 (16.4) | 34 (17.2) | 0.9(0.42-2.11) 0.89 |
|  |  |  |  |
| **Indications, n (%)**  Positive fecal occult blood test  Postpolypectomy surveillance  Change in bowel habits  Anemia  Rectal bleeding  Familial screening  Others | 13 (23.6)  14 (5.5)  15 (9.1)  12 (21.8)  6 (10.9)  1 (1.8)  4 (7.3) | 48 (24.2)  54 (27.3)  19 (9.6)  36 (18.2)  19 (9.6)  12 (6.1)  10 (5.1) | 0.96(0.48-1.95) 0.36  0.9(0.46-1.80) 0.87  0.9(0.34-2.65) 0.22  1.26(0.60-2.62) 0.92  1.2 (0.44-3.05) 0.12  0.3 (0.04-2.26) 0.37  1.5 (0.44-4.90) 0.55 |
| **Comorbidity, n (%)** |  |  |  |
| Diabetes | 48 (87.3) | 152 (76.8) | 2.1 (0.88-4.90) 0.09 |
| Stroke | 8 (14.5) | 10 (5.1) | 3.2 (1.20-8.55) 0.02 |
| Cirrhosis | 1 (1.8) | 4 (2) | 1.1 (0.12-10.17) 0.92 |
| Chronic renal failure  Hypertension | 6 (11.1)  24 (43.6) | 27 (13.6)  77 (38.9) | 0.8 (0.31-2.03) 0.63  1.2 (0.66-2.23) 0.53 |
| Constipation | 16 (29.1) | 31 (15.7) | 2.2 (1.10-4.44) 0.02 |
| Abdominal/pelvic surgery | 29 (52.7) | 89 (44.9) | 1.4 (0.75-2.49) 0.3 |
| Medical treatment, n (%) |  |  |  |
| Opioids | 7 (12.7) | 11 (5.6) | 2.5 (0.91-6.76) 0.07 |
| Calcium antagonists | 13 (23.6) | 31 (15.7) | 1.7 (0.80-3.46) 0.17 |
| Antidepressants | 3 (5.5) | 9 (4.5) | 1.2 (0.32-4.63) 0.78 |
| Bowel preparation adherence, n (%) | 2 (3.6) | 0 | 0.96 (0.92-1.01) 0.05 |
| 4 L PEG, n (%) | 26 (47.3) | 102 (51.5) | 0.8 (0.46-1.54) 0.58 |
| Dissatisfaction with the bowel preparation, n (%) | 10 (18.2) | 19 (9.6) | 2.1 (0.91-4.81) 0.08 |
| Difficulties following the bowel preparation, n (%) | 3 (5.5) | 1 (0.5) | 11.4 (1.16-111.10) 0.03 |
| Adverse effects, n (%) | 28 (21.9) | 23 (18.4) | 1.2 (0.67-2.30) 0.49 |

^a^BBPS<2: Boston bowel preparation scale < 2 points per segment

^b^BBPS≥2: Boston bowel preparation scale **≥** 2 points per segment

^c^Body mass index

^d^Education higher than high school

^e^First-degree relatives with colorectal cancer
