# Supplementary material for: An Enhanced High-Volume Preparation for Colonoscopy Is Not Better Than a Conventional Low-Volume One in Patients at Risk of Poor Bowel Cleansing: A Randomized Controlled Trial
Source: Front Med (Lausanne). 2021 Mar 22;8:654847. doi: 10.3389/fmed.2021.654847 (PMC8019748; doi:10.3389/fmed.2021.654847)
Supplement: Supplementary file 3 [file Table_2.doc]

Supplementary table 2.

| **Variables** | **OR^a^ (95% CI^b^)** | **P** |
| --- | --- | --- |
| Age | 0.97 (0.934-1.001) | 0.06 |
| Stroke  (Ref: no stroke) | 3.22 (1.174-8.848) | 0.02 |
| Difficulties following the preparation  (Ref: no difficulties) | 12.06 (1.151-126.295) | 0.04 |
| Constipation  (Ref: no constipation) | 1.87 (0.896-3.888) | 0.1 |

^a^ Odds ratio

^b^ Confidence interval

^c^ Reference category
